# Oral vinorelbine and continuous low doses of cyclophosphamide in pediatric rhabdomyosarcoma: a real-world study

**DOI:** 10.3389/fphar.2023.1132219

**Published:** 2023-05-03

**Authors:** Yingxia Lan, Liuhong Wu, Ye Hong, Xiaofei Sun, Juan Wang, Junting Huang, Feifei Sun, Jia Zhu, Zijun Zhen, Yizhuo Zhang, Mengjia Song, Suying Lu

**Affiliations:** ^1^ Sun Yat-sen University Cancer Center, State Key Laboratory of Oncology in South China, Collaborative Innovation Center for Cancer Medicine, Guangzhou, China; ^2^ Department of Pediatric Oncology, Sun Yat-sen University Cancer Center, Guangzhou, China

**Keywords:** pediatric, rhabdomyosarcoma, metronomic maintenance therapy, soft tissue sarcoma, chemotherapy

## Abstract

**Introduction:** Metronomic maintenance therapy (MMT) has significantly improved the survival of patients with high-risk rhabdomyosarcoma in clinical trials. However, there remains a lack of relevant data on its effectiveness in real-world situations.

**Methods:** We retrospectively retrieved data of 459 patients < 18 years of age diagnosed with rhabdomyosarcoma at Sun Yat-sen University Cancer Center from January 2011 to July 2020 from our database. The MMT regimen was oral vinorelbine 25–40 mg/m^2^ for twelve 4-week cycles on days 1, 8, and 15, and oral cyclophosphamide 25–50 mg/m^2^ daily for 48 consecutive weeks.

**Results:** A total of 57 patients who underwent MMT were included in the analysis. The median follow-up time was 27.8 (range: 2.9–117.5) months. From MMT to the end of follow-up, the 3-year PFS and OS rates were 40.6% ± 6.8% and 58.3% ± 7.2%, respectively. The 3-year PFS was 43.6% ± 11.3% in patients who were initially diagnosed as low- and intermediate-risk but relapsed after comprehensive treatment (20/57), compared with 27.8% ± 10.4% in high-risk patients (20/57) and 52.8% ± 13.3% in intermediate-risk patients who did not relapse (17/57). The corresponding 3-year OS for these three groups was 65.8% ± 11.4%, 50.1% ± 12.9%, and 55.6% ± 13.6%, respectively.

**Conclusion:** We present a novel study of MMT with oral vinorelbine and continuous low doses of cyclophosphamide in real-world pediatric patients with RMS. Our findings showed that the MMT strategy significantly improved patient outcomes and may be an effective treatment for high-risk and relapsed patients.

## 1 Introduction

Rhabdomyosarcoma (RMS), which represents a high-grade neoplasm in which cancer cells have a propensity for myogenic differentiation, is the most common soft tissue sarcoma in children and adolescents (Skapek et al., 2019). Over the last 3 decades, despite many advances with comprehensive treatment strategies involving multiple disciplines, such as chemotherapy, surgery, and radiotherapy, the chance of cure for children with recurrent and widely metastatic disease remains very low (Yohe et al., 2019). According to the risk stratification of Children’s Oncology Group (COG) (Crane et al., 2022), the overall survival rate can exceed 90% among low-risk patients, approximately 70% among intermediate-risk patients, but only less than 30% among high-risk patients. Moreover, multidrug combinations or the addition of targeted therapy did not significantly improve the survival of patients with high risk and patients who were low or intermediate risk at diagnosis but had refractory or relapsed disease (Haduong et al., 2022).

Vinorelbine (VNR) has been confirmed as an effective treatment in previously treated advanced childhood sarcomas (Casanova et al., 2022). VNR and continuous low doses cyclophosphamide (CTX) showed a good response rate in relapsed, refractory, or metastatic RMS (Casanova et al., 2004; Klingebiel et al., 2008; Minard-Colin et al., 2012). Interestingly, a randomized trial revealed that the addition of metronomic maintenance therapy (MMT) with VNR plus CTX for children with high-risk RMS resulted in a significant increase in overall and event-free survival in the European paediatric Soft tissue sarcoma Study Group (EpSSG) (Bisogno et al., 2019). The introduction of maintenance chemotherapy included six cycles of intravenous VNR 25 mg/m^2^ on days 1, 8, and 15, and daily oral CTX 25 mg/m^2^ on days 1–28 (Bisogno et al., 2019). Patients at a high risk of relapse had a 5-year overall survival (OS) rate of 86.5% in the MMT group with manageable toxicity compared with 73.3% in the non-MMT group.

In fact, the detailed clinical application of MMT in RMS still needs to be discussed. First, more convenient drug preparations should be selected. Intravenous VNR has demonstrated encouraging results in RMS. Oral VNR appears to be a more convenient and more economical attractive candidate for the management of RMS. Previous studies have shown that oral VNR has the same pharmacokinetic-pharmacodynamic relationship as intravenous VNR (Gebbia and Puozzo, 2005). In our study, one major improvement in maintenance therapy would be the use of oral VNR instead of intravenous VNR. Second, the duration of MMT is another crucial issue. Although the duration of maintenance therapy for high-risk RMS patients was 6 months (Bisogno et al., 2019), the follow-up EpSSG FaR-RMS trial (EudraCT Number: 2018-000515-24) is investigating the role of a longer duration of MMT with CTX and VNR (randomization 6 vs. 12 months). Randomization of between 1 and 2 years of maintenance with this MMT has also been proposed for stage IV RMS. Third, which group of patients can benefit more from MMT needs to be further studied. Until now, the role of MMT has not been studied in patients who were at low and intermediate-risk at diagnosis but relapsed or had small residual lesions at the end of treatment. Apart from high-risk patients, these patients also have a high risk of recurrence. Therefore, these patients may also benefit from maintenance treatment. Additionally, finding the right dose remains important for the successful use of MMT.

In the present study, we used oral VNR instead of intravenous VNR for a longer duration of maintenance with 1 year. We evaluated the efficacy of MMT not only in RMS patients with high risk but also in those with low and intermediate-risk at diagnosis but relapsing or with small residual lesions at the end of comprehensive treatment.

## 2 Patients and methods

### 2.1 Study population

Patients < 18 years of age diagnosed with RMS at Sun Yat-sen University Cancer Center from January 2011 to July 2020 were retrospectively identified from our database. Patients who were lost to follow-up after initial examination and treatment were excluded. The inclusion criteria of patients undergoing MMT were as follows: 1) First relapsed patients achieved clinical complete remission (cCR) or complete remission (CR) after comprehensive treatment. cCR was defined as patients who had residual lesions but with no fluorodeoxyglucose metabolism detected by PET/CT; 2) intermediate-risk patients who achieved cCR or patients with Intergroup Rhabdomyosarcoma Study (IRS) stage III who did not receive radiotherapy; and 3) high-risk patients who achieved CR or cCR after standard treatment were assigned to continue maintenance chemotherapy. Patients’ data were followed up by telephone and access to outpatient and inpatient data. Patient follow-up was current through 31 December 2021. This study was approved by the Ethics Committee of Sun Yat-sen University Cancer Center (Approval Number: B2022-489-01) and conducted according to the latest version of the Declaration of Helsinki. The requirement for informed consent was waived by the institutional review committee.

### 2.2 Risk stratification

Risk stratification for RMS is based on a pretreatment Tumor Node Metastasis (TNM) staging system and surgical/pathologic clinical grouping system. Patients were divided into low-risk, intermediate-risk, and high-risk groups according to COG risk stratifications (Rudzinski et al., 2015). In our study, patients in low-risk Subset A and Subset B were included in the low-risk group.

### 2.3 Treatment protocol

All patients received chemotherapy, surgery, and/or local radiotherapy, followed by MMT (Table 1). Chemotherapy regimens were administered alternately at 3-week intervals. Patients who relapsed were given multiple cycles of chemotherapy with different drug combinations proven to be effective at present in combination with surgery (if surgical resection was possible after assessment by the surgeon) and radiotherapy (if radiotherapy was possible after assessment by the radiologist), followed by MMT after cCR or CR was achieved.

**TABLE 1:** Chemotherapy regimens for RMS.

Chemotherapy regimens	Drugs dosage and administration
Low-risk group	VCR1.5 mg/m^2^/d (≯2 mg), iv, d1
VAC 8 cycles	ACT-D 45 ug/kg/d (≯2,500 ug), iv drip, d1 CTX 1.2 g/m^2^/d, iv drip, d1
Intermediate-risk group	CTX 1.0 g/m^2^/d, iv drip, d1
CAV	THP 50 mg/m^2^/d, iv, d1
(Cycle 1, 3, 5, 7, 9)	VCR1.5 mg/m^2^/d (≯2 mg), iv, d1
I.E.,	IFO 1.5 g/m^2^/d, iv drip, d1-5
(Cycle 2, 4, 6, 8, 10)	Etoposide 100 mg/m^2^/d, iv drip, d1-5
High-risk group	CTX 1.0 g/m^2^/d, iv drip, d1-2
CAV	THP 50 mg/m^2^/d, iv, d1
(Cycle 1, 3, 5, 7, 9, 11 and 13)	VCR1.5 mg/m^2^/d (≯2 mg), iv, d1
I.E.,	IFO 1.8 g/m^2^/d, iv drip, d1-5
(Cycle 2, 4, 6, 8, 10, 12 and 14)	Etoposide 100 mg/m^2^/d, iv drip, d1-5
During radiotherapy^a^	VCR1.5 mg/m^2^/d (≯2 mg), iv, d1
VI 2 cycles	Irinotecan 50 mg/m^2^/d, iv drip, d1-5

^a^ During radiotherapy, the VI, regimen was administered concurrently for sensitization, and 2 cycles of VI, were not included in the total cycles of treatment.

Mesna will be used with cyclophosphamide and ifosfamide.

VAC: vincristine (VCR), actinomycin-D (Act-D), and cyclophosphamide (CTX); CAV: cyclophosphamide, pirarubicin (THP), and vincristine; I.E.: ifosfamide (IFO), and etoposide; VI: vincristine, irinotecan.

Despite the reliance on low doses, right dosing remains important for successful use of MMT. A minimum level of exposure to anticancer agents is essential to obtain a meaningful clinical effect. Considering the convenience of drug use for children, we have given a dosage selection range to facilitate the cutting and rounding of tablet or capsule drugs. The MMT regimen was oral VNR 25–40 mg/m^2^ for twelve 4-week cycles on days 1, 8, and 15, and oral CTX 25–50 mg/m^2^ daily for 48 consecutive weeks. Patients in the intermediate-risk and high-risk groups generally underwent surgery in the 10th week and radiotherapy in the 16th week; however, radiotherapy within the 12th week was considered for high-risk patients with parameningeal lesions or central system involvement. The starting time of MMT was the time when peripheral blood leukocytes reached 3×10^9^/L or neutrophils reached 1×10^9^/L after the end of treatment. The duration of MMT was 48 weeks (if tolerated) or until disease progression, relapse, or metastasis. Disease assessment was performed by computed tomography or magnetic resonance imaging every 3 months during MMT.

### 2.4 Statistical analysis

Progression-free survival (PFS) was defined as the time from the start of MMT to the occurrence of disease progression or all-cause death or time of last follow-up if no event had occurred. OS was defined as the time from the start of MMT to all-cause death or last follow-up. PFS and OS were censored at the date of the last follow-up visit. Survival analysis was performed using the Kaplan-Meier method, and data were compared using the log-rank test. Statistical analyses were performed by SPSS version 26.0 and GraphPad Prism 9.0. A *p*-value of <0.05 was considered statistically significant.

## 3 Results

### 3.1 Patient characteristics

Between January 2011 and July 2020, a total of 459 patients with RMS were treated in our center, and 57 patients (12.4%) undergoing MMT were eventually included in the analysis. The patients’ characteristics are summarized in Table 2. The median age of the patients was 6.6 years (range: 0.2–17.9 years). The male-to-female ratio was 1.38:1.0. ERMS was the main pathological type (70.2%). The most common primary sites were retroperitoneal (26.3%), parameningeal (24.6%), trunk (19.3%), and extremities (14.0%). Of the 459 patients, 163 were at low risk, 170 were at intermediate risk, and 124 were at high risk. Among 57 patients undergoing MMT, 14 cases were low risk at initial diagnosis but relapsed, 23 cases were intermediate risk, and 20 cases were high risk at diagnosis. Among the 20 high-risk patients receiving MMT, the most common sites of initial metastasis were lymph nodes (10/20), multiple bones (8/20), lung and bone marrow (4/20), liver (3/20), abdominal pelvis cavity (2/20), pancreas (2/20), bladder (1/20), kidney (1/20), testis (1/20), and adrenal gland (1/20).

**TABLE 2:** Patient characteristics.

Characteristic	n (%)
Sex	
Female	24 (42.1)
Male	33 (57.9)
Age at diagnosis, year	
≤1	3 (5.3)
1–9	39 (68.4)
≥10	15 (26.3)
Histology	
Alveolar	14 (24.6)
Embryonal	40 (70.2)
Spindle cell/sclerosing	0 (0)
Pleomorphic	0 (0)
NOS/unknown	3 (5.3)
FOXO1 fusion status	
Fusion-positive	3 (21.4)
Unknown	11 (78.6)
Primary size	
≤5 cm	24 (42.1)
>5 cm	25 (43.9)
Unknown	8 (14.0)
Tumor site	
Extremity	8 (14.0)
Parameningeal	14 (24.6)
Bladder/prostate	1 (1.8)
Testicle	2 (3.5)
Head and neck	4 (7.0)
Retroperitoneal	15 (26.3)
Trunk	11 (19.3)
Orbit	2 (3.5)
TNM staging	
1	7 (12.3)
2	9 (15.8)
3	25 (43.9)
4	16 (28.1)
IRS staging	
I	5 (8.8)
II	9 (15.8)
III	23 (40.4)
IV	20 (35.1)
Number of metastatic sites	
0	37 (64.9)
1	10 (17.5)
2	4 (7.0)
3	5 (8.8)
4	1 (1.8)
Radiation therapy techniques	
3DCRT	4 (7.0)
IMRT	24 (42.1)
VMAT	6 (10.5)
TOMO	7 (12.3)

NOS, not otherwise specified; TNM, tumor node metastasis; IRS, intergroup rhabdomyosarcoma study; 3DCRT, three-dimensional conformal radiation therapy; IMRT, intensity modulated radiation therapy; VMAT, volumetric modulated arc therapy.

Among the 14 patients who were low risk at initial diagnosis receiving MMT, six cases relapsed after comprehensive treatment, and eight cases relapsed without treatment after initial surgical resection. Most of the intermediate-risk patients receiving MMT were those with a poor response to standard treatment, among whom 12 patients achieved cCR at the end of comprehensive treatment, one patient relapsed without treatment after surgery, five patients relapsed after comprehensive treatment, and five patients with IRS stage III did not receive radiotherapy. In high-risk patients, MMT was generally considered. However, MMT was not administered to all high-risk patients, especially those with poor chemotherapy tolerance. Additionally, MMT is not currently recognized as part of standard care, so the preferences of physicians and patients greatly influence the selection of high-risk patients. All patients achieved cCR or CR after comprehensive therapies at the time of enrollment.

### 3.2 Treatment outcome

**FIGURE 1:**
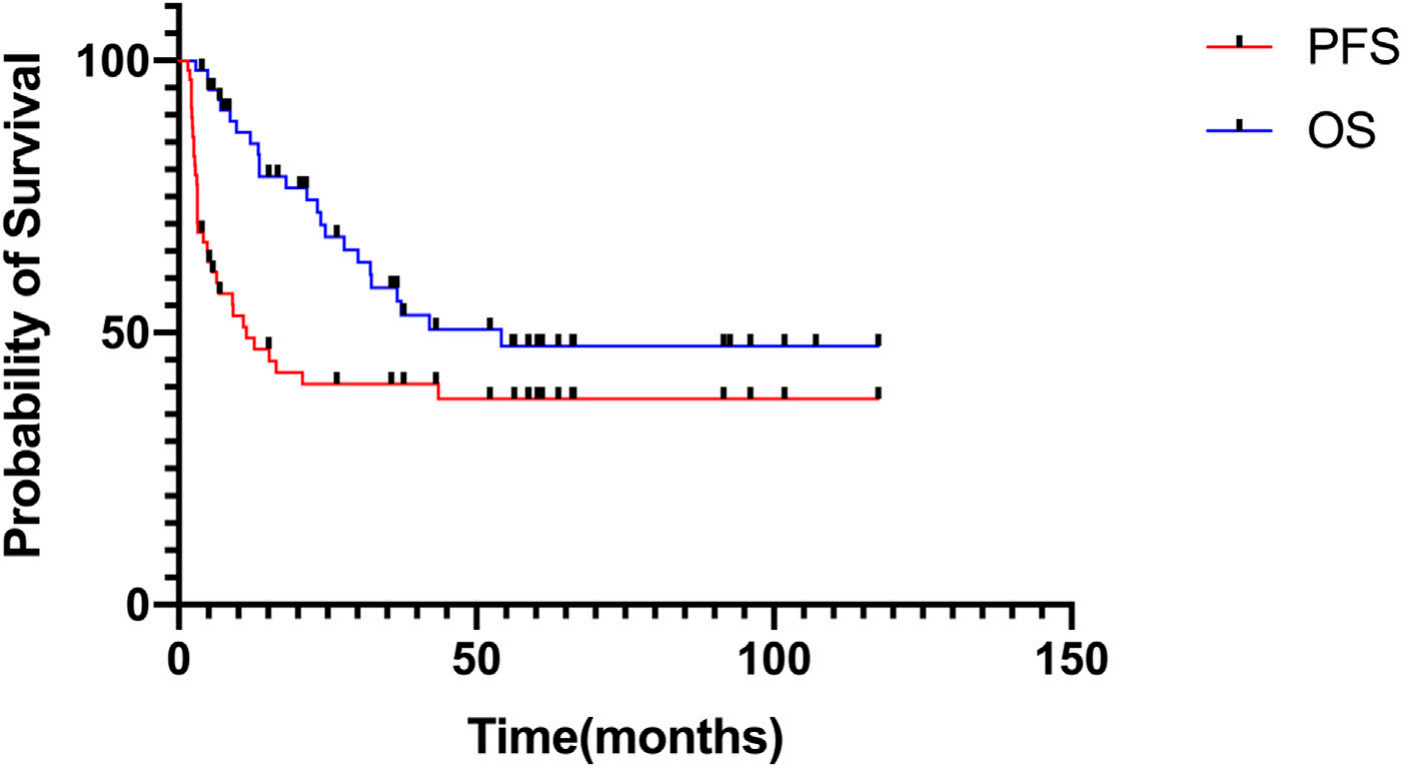
Progression-free survival and overall survival of patients receiving MMT in the entire cohort.

The median follow-up time was 27.8 months (range: 2.9–117.5 months). In the entire cohort, the 3-year PFS and OS rates were 40.6% ± 6.8%, and 58.3% ± 7.2%, respectively; the 5-year PFS and OS rates were 37.9% ± 6.9%, and 47.6% ± 7.7%, respectively (Figure 1). The median duration of MMT was 4 months (range: 1–36 months). There was an extension of treatment duration in four patients following their wishes, and the total treatment duration in the patients was 14, 17, 22, and 36 months.

The disease status of patients at the time of MMT administration was CR (*n* = 24), cCR (*n* = 33). At the end of MMT, 11 of the 24 patients with CR had progressive disease (PD), and 13 patients maintained CR. Of the remaining 33 patients with cCR, four patients had CR, seven patients had cCR, and 22 patients had PD, including one patient who developed a second tumor.

After MMT, 4 of the 33 patients with PD died without additional treatment. Among the 29 treated patients, 20 received chemotherapy alone, two received chemotherapy and surgery, four received chemotherapy, surgery, and radiotherapy, and three received chemotherapy and radiotherapy. After MMT, 12 patients developed metastasis, and the most common site of metastasis was the lungs (3/12).

**FIGURE 2:**
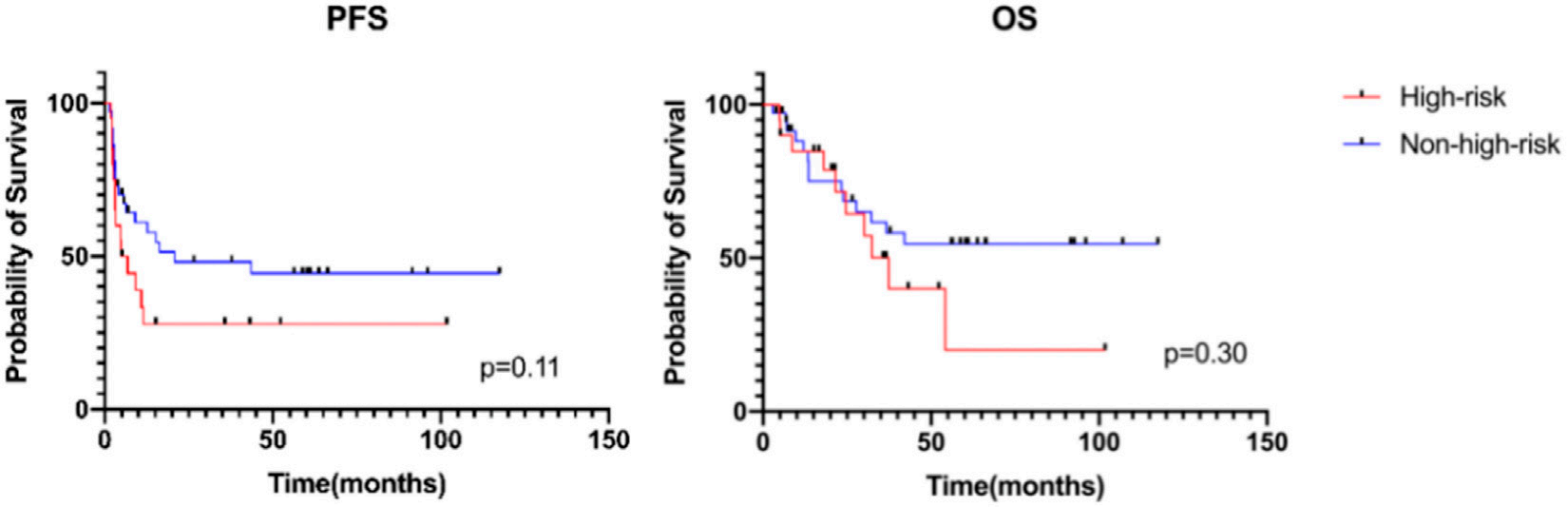
Progression-free survival and overall survival of patients receiving MMT between different risk groups.

Among the 57 patients receiving MMT, we compared the survival of high-risk patients with that of non-high-risk patients at diagnosis. Non-high-risk patients were defined as low- and intermediate-risk patients at diagnosis who relapsed after comprehensive treatment, intermediate-risk patients who achieved cCR or patients with IRS stage III who did not receive radiotherapy. The 3-year PFS was 27.8% ± 10.4% in high-risk patients versus 48.1% ± 8.6% in non-high-risk patients [hazard ratio (HR) 1.73 (95% CI 0.82–3.63); *p* = 0.11], and the 3-year OS was 50.1% ± 12.9% in high-risk patients versus 61.6% ± 8.8% in non-high-risk patients [HR 1.52 (95% CI 0.64–3.58); *p* = 0.30]. The 5-year PFS was 27.8% ± 10.4% in high-risk patients versus 44.4% ± 8.7% in non-high-risk patients, and the 5-year OS was 20.0% ± 15.7% in high-risk patients versus 54.6% ± 9.1% in non-high-risk patients (Figure 2).

**FIGURE 3:**
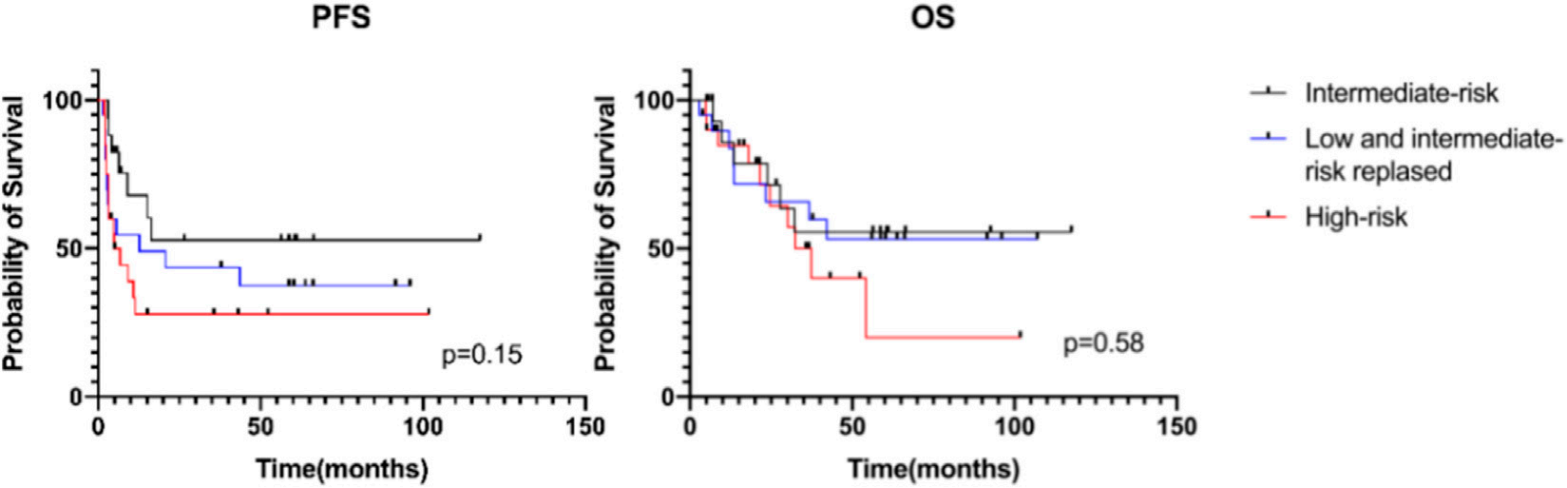
Progression-free survival and overall survival of patients receiving MMT by risk group stratum.

We further analyzed the survival of low- and intermediate-risk patients at diagnosis who relapsed and found that the 3-year PFS was 43.6% ± 11.3% in these patients (20/57), compared with 27.8% ± 10.4% in high-risk patients (20/57) and 52.8% ± 13.3% in intermediate-risk patients who did not relapse (17/57). The corresponding 3-year OS for these three groups was 65.8% ± 11.4%, 50.1% ± 12.9%, and 55.6% ± 13.6%, respectively. The corresponding 5-year PFS for these three groups was 37.4% ± 11.3%, 27.8% ± 10.4%, and 52.8% ± 13.3% (*p* = 0.15). The 5-year OS for these three groups was 53.2% ± 12.3%, 20.0% ± 15.7%, and 55.6% ± 13.6% (*p* = 0.58). However, there was no significant difference between the relapsed group and the other two groups (Figure 3).

## 4 Discussion

Current studies on MMT are limited to a few clinical trials showing that administration of MMT after standard therapy improves the overall survival of RMS patients (Casanova et al., 2004; Klingebiel et al., 2008; Minard-Colin et al., 2012; Bisogno et al., 2019; Koscielniak et al., 2022). In the present study, we included nearly 10 years of pediatric RMS patients receiving MMT and found that their OS in the real world was slightly lower than what has been observed in the largest MMT clinical trials (Bisogno et al., 2019) but better than the current COG study in high-risk and relapse/refractory patients without MMT (Haduong et al., 2022). That may be because the risk stratification in the RMS 2005 study (Bisogno et al., 2019) was different from that of the COG study (Crane et al., 2022), and its definition of high-risk patients was similar to that of the intermediate-risk patients in our study, resulting in a higher 5-year OS of 86.5% after standard treatment and maintenance therapy. Based on a similar risk stratification study of patients with metastatic RMS receiving maintenance therapy (Schoot et al., 2022), the PFS and OS was similar to our patients, with improved outcomes compared to historical cohorts. Therefore, our study is similar to previous MMT studies (Casanova et al., 2004; Klingebiel et al., 2008; Minard-Colin et al., 2012; Koscielniak et al., 2022; Schoot et al., 2022), showing the effectiveness of MMT in relapsed and high-risk RMS patients. In the absence of a clear application method and indication of MMT in RMS at present, our study has the following highlights. Most importantly, VNR was administered orally rather than intravenously, which was more convenient for children than previous studies (Casanova et al., 2004; Klingebiel et al., 2008; Minard-Colin et al., 2012; Bisogno et al., 2019) and did not affect the final survival. Furthermore, we extended the use population of MMT in RMS. We included not only high-risk patients but also relapsed patients after standard treatment or those who had a high risk of recurrence. Another crucial issue relies on the duration of treatment. The duration of maintenance therapy for high-risk patients with RMS was 6 months (Bisogno et al., 2019). However, in the follow-up European EpSSG protocol, a 1-year duration design has already been proposed for patients with high-risk RMS. Randomization of the duration of maintenance of 1 or 2 years has also been proposed for stage IV RMS. Moreover, in EpSSG’s MTS 2008 study (Schoot et al., 2022), metastatic patients treated with MMT for up to 1 year delayed the median time from random assignment to relapse from 6.9 months to 10.1 months, with the majority of events taking place after the 24-week window for maintenance treatment. Therefore, we adopted 48 weeks of MMT in the expectation for better disease control, which provides available data for a longer duration of maintenance treatment. Therefore, our data provide a new perspective basis for further clinical application of MMT in the future to improve the survival of RMS patients, especially those with poor prognosis. It may be possible to expand the use of MMT in patients who relapsed or achieved cCR after comprehensive therapy.

Given the availability of oral drugs and convenience of medication for patients, VNR was changed from intravenous administration to oral administration in this study. Compared with the intravenous route, oral anticancer drugs have many advantages, especially the reduction of local intravenous toxicity at the injection site and the overall improvement of patient convenience and quality of life. It is also a favorable choice in long-term medication management of disease. For VNR, oral administration had the same pharmacokinetic-pharmacodynamic relationship as intravenous administration (Gebbia and Puozzo, 2005). Oral dosing in children is challenged by VNR liquid capsule formulation and CTX tablet limitations. Therefore, in our study, the doses of VNR and CTX fluctuated and were rounded on the basis of VNR 40 mg/m^2^ and CTX 25–50 mg/m^2^. Repeated administration of VNR at the same dose and frequency in pediatric patients has been shown to produce similar systemic exposures (Hamimed et al., 2022). Therefore, it is believed that oral VNR can be used to improve outcomes and quality of life in patients with RMS.

The oral dose of VNR is usually 60 mg/m^2^; however, too-high doses of VNR can cause protumoral host responses and prevent desired effects (Shaked et al., 2019). Therefore, we tried a lower dose of VNR of 25∼40 mg/m^2^. It seems it does not lower the efficacy which would be consistent with a metronomic-based mechanisms of action. The dosage of CTX in metronomic therapy varies in different studies. CTX (30 mg/m^2^ PO daily) was continually given in pediatric recurrent solid tumors (Stempak et al., 2006). Another study showed that CTX was given at 40 mg/m^2^/day (PO) combined with vinblastine in patients with desmoplastic small round cell tumors, which was correlated with prolonged time to relapse (Scheer et al., 2019). A higher dose of CTX (2.5 mg/kg/day PO) (Kieran et al., 2005), etoposide, temozolomide, in combination with alternating cytostatic biologic therapy, celecoxib and isotretinoin were studied in patients with malignant central nervous system tumors (Choi et al., 2008). In fact, the right dose to ensure efficient and low toxicity is a critical issue that needs to be further studied.

The standard treatment regimen at our center was modified based on current COG studies. We adopted the VAC (vincristine/actinomycin-D/cyclophosphamide) regimen every 3 weeks in the low-risk group, which was similar to the Intergroup Rhabdomyosarcoma Study-IV (IRS-IV) (Crist et al., 2001), D9602 (Raney et al., 2011), and ARST0331 (Walterhouse et al., 2014). ARST0331 achieved good clinical results, reducing toxicity without affecting OS and simplifying the treatment of low-risk patients. The regimen in the present study was similar to those in the above studies, although the total dose of VCR was reduced and the cumulative CTX dose (9.6 g/m^2^) was doubled compared with that of ARST0331 (4.8 g/m^2^). VDC/I.E., has been proven to be as effective for intermediate-risk RMS as IRS-IV (Arndt et al., 2008). Considering the large cumulative dose of CTX in D9803 (Arndt et al., 2009), ifosfamide was used to replace CTX to prevent losing the therapeutic effect, and the CAV/IE regimen was used alternately for 10 cycles in the intermediate-risk group in this study. Considering the effectiveness recorded in the ARST0531 study (Hawkins et al., 2018), it was changed to the VI regimen for 2 cycles during radiotherapy. In this study, the chemotherapy regimen in high-risk patients was similar to COG’s intensive multiagent therapy (Weigel et al., 2016). A CAV/IE regimen was used every 3 weeks for 14 cycles and 2 cycles of the VI regimen during radiotherapy, in which doxorubicin was replaced with pirarubicin owing to its cardiotoxic effects (Dantchev et al., 1979).

We found that the PFS and OS of high-risk patients at diagnosis were worse than those of non-high-risk patients at diagnosis; however, there was no significant difference between the two groups, which may be explained by the fact that most of the non-high-risk patients at diagnosis included in this study were relapsed patients or those who had a high risk of recurrence. Additionally, we further grouped metastatic patients receiving MMT according to Oberlin prognostic factors (Oberlin et al., 2008) and found that the 5-year PFS was 15.4% ± 10% in patients with two or more Oberlin risk factors versus 57.1% ± 18.7% in patients with one or no risk factors [HR 2.12 (95% CI 0.71–6.28); *p* = 0.23], and the 5-year OS was 26.9% ± 15.7% in patients with two or more Oberlin risk factors versus 66.7% ± 19.2% in patients with one or no risk factors [HR 3.58 (95% CI 1.04–12.4); *p* = 0.08]. Both PFS and OS in patients with two or more Oberlin risk factors were lower than those with one or no risk factor (Supplementary Figure S1), suggesting the feasibility of Oberlin prognostic factors in metastatic patients receiving MMT in the real world.

There is currently no universal standard regimen for relapsed patients with RMS. Some studies have found that the prognosis of non-metastatic relapsed patients depends on several related factors, such as radiotherapy, tumor size, and intensity of treatment, and the survival rate varies from 2% to 60% (Chisholm et al., 2011; Affinita et al., 2020; Heske et al., 2021). However, we found that the 3-year OS of non-metastatic relapsed patients was approximately 70%, higher than that of previous studies, indicating that MMT may be a new and effective standard of care in patients with non-metastatic relapsed RMS. MMT is mostly used in high-risk patients in clinical trials and has not been used in low- and intermediate-risk patients; therefore, the present study provides a new perspective that the use of MMT after salvage therapy in non-metastatic relapsed patients can significantly improve the outcome.

Previous studies showed that in group III participants for IRS-IV, the response at the end of treatment was not associated with disease recurrence or death, resection of the residual mass was not associated with improved prognosis, and aggressive alternative therapy may not be warranted (Rodeberg et al., 2009). However, in this study, when patients received MMT after the end of treatment, 11 of 24 patients with CR progressed, compared with 22 of the remaining 33 patients with cCR. Given the benefit of MMT, nearly 70% of patients with cCR progressed even when patients received MMT. Thus, for patients who failed to achieve a CR at the end of treatment, MMT was one of the recommended therapies (André et al., 2020), a strategy that needs to be further confirmed in future clinical trials.

In terms of toxicity, MMT with oral VNR and continuous low doses of CTX was generally safe, with no treatment-related deaths. No grade 3 or 4 toxic events were observed. In addition, bone marrow suppression rarely occurs during MMT because of regular monitoring of routine blood tests and adjustment of the dose of oral drugs according to the results of routine blood tests. Although some patients occasionally had mild gastrointestinal symptoms, there was no need to go to the hospital for treatment of adverse reactions.

This study had some limitations. On the one hand, this was a retrospective study with patient selection bias, and the sample size was relatively small. On the other hand, as not all patients were examined for FOXO1 fusion genes, the latest risk grouping based on positive/negative fusion genes was limited.

In conclusion, our study showed that MMT with oral VNR and continuous low doses of CTX are effective and feasible for pediatric patients with RMS in the real world. This treatment could be further studied in patients with high-risk and relapsed RMS in prospective clinical trials.

## Data Availability

The original contributions presented in the study are included in the article/Supplementary Material, further inquiries can be directed to the corresponding author.
