# Supplementary figures and images for: Oral vinorelbine and continuous low doses of cyclophosphamide in pediatric rhabdomyosarcoma: a real-world study
Source: Front Pharmacol. 2023 May 3;14:1132219. doi: 10.3389/fphar.2023.1132219 (PMC10188979; doi:10.3389/fphar.2023.1132219)

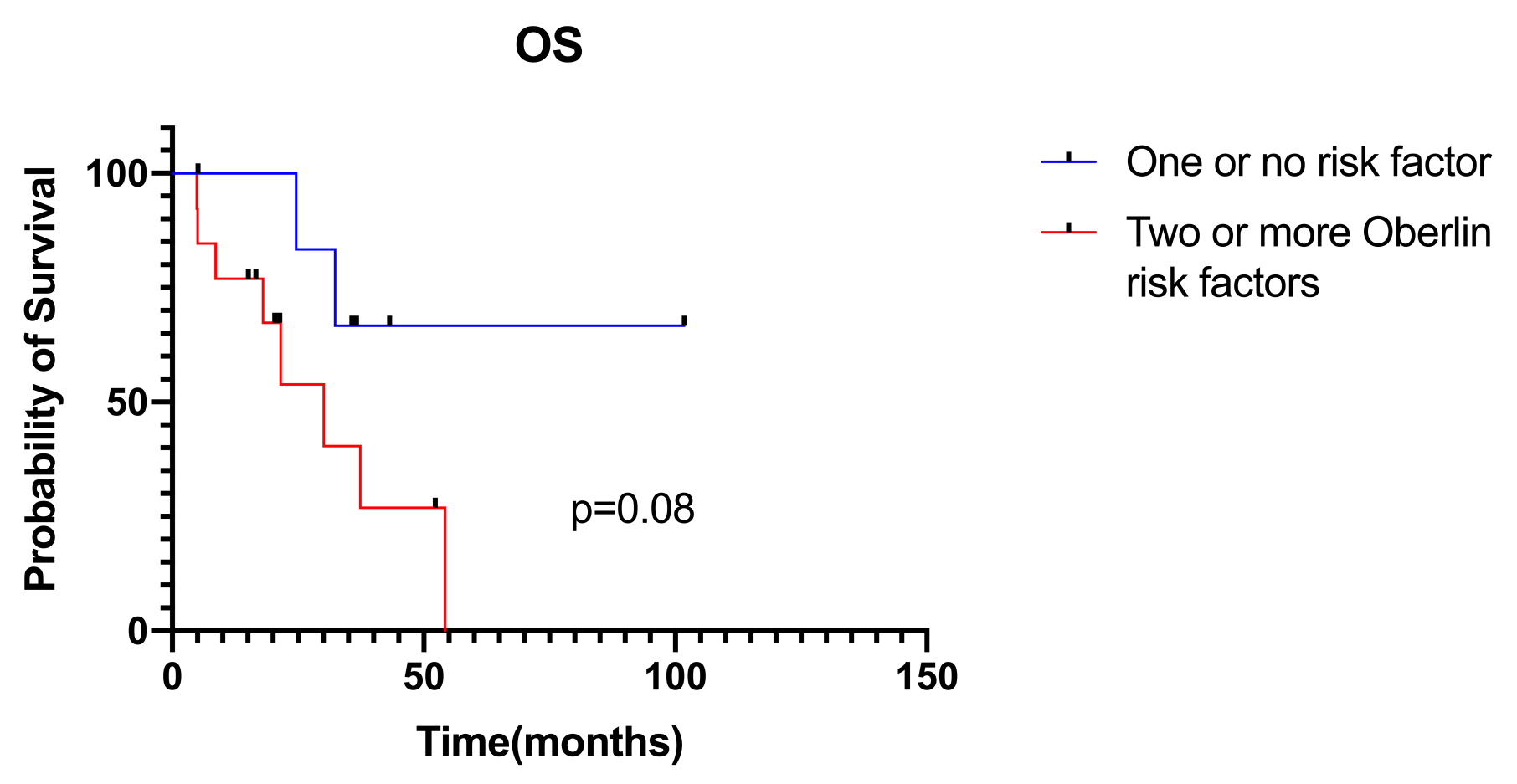

Supplement: Supplementary file 1 [file Image2.TIF]

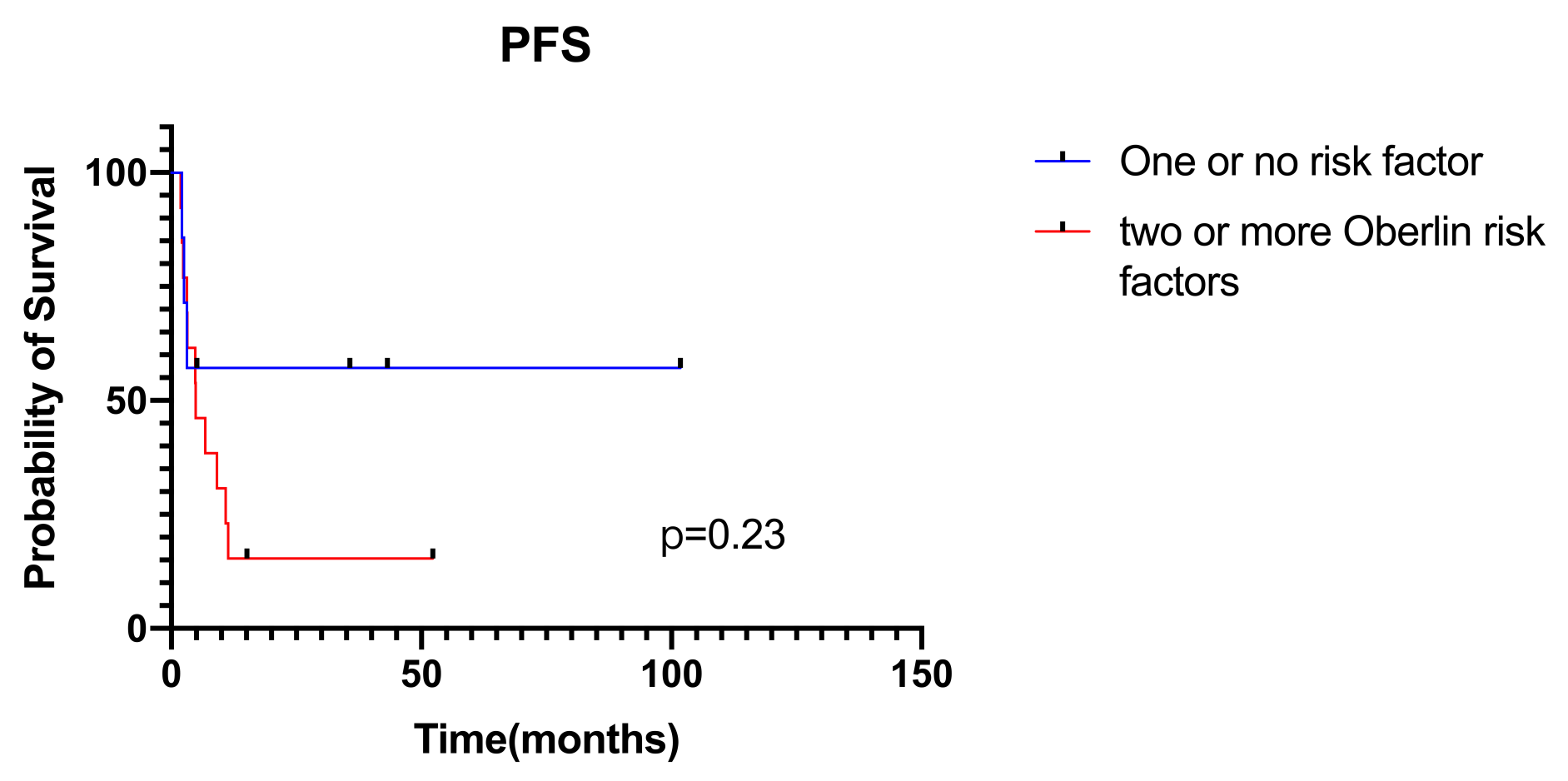

Supplement: Supplementary file 2 [file Image1.TIF]
